# Weighing the Benefits of Fish Oil for Patients With Prostate Cancer: A Subcohort Review From the SELECT Trial

**DOI:** 10.6004/jadpro.2015.6.4.8

**Published:** 2015-07-01

**Authors:** Marilyn Haas-Haseman

**Affiliations:** Carolina Clinical Consultant, Arden, North Carolina

Many individuals, both men and women, seek dietary supplements with the belief that they will protect them from developing certain chronic diseases and possibly even decrease their risk of developing cancer (National Cancer Institute, 2015). Fish oil is of the most popular dietary supplements, containing two of the most important omega-3 acids: eicosapentaenoic acid (EPA) and docosahexaenoic acid (DHA). Humans cannot produce their own omega-3 fatty acids or make them from omega-6 fatty acids. Therefore, external intake of these acids is important to one’s health.

Two external means of obtaining omega-3 fatty acids in diet are eating cold water fish such as salmon, sardines, herring, mackerel, tuna, or anchovy or taking fish oil supplements. Sometimes, oral fish oil supplements contain tiny amounts of vitamin E to prevent spoilage and other minerals such as calcium and iron, or they are orally mixed with vitamins A, B₁, B₂, B₃, C, or D. It is necessary for patients and advanced practitioners to read supplement labels. Also, preparation of fish (broil or baked, not fried) is important to obtain the maximal benefit of the omega-3 fatty acids.

Fish oil has long been taken for various conditions. Scientific evidence suggests that omega-3 fatty acids can reduce pain, swelling, and inflammation (Goldberg & Katz, 2007; Maroon & Bost, 2006). Strong evidence supports the idea that fish oil helps to lower high-density proteins and the risk of heart disease and stroke (Natural Medicines Comprehensive Database, 2015). There is ongoing research on the use of fish oil for other purposes.

## FISH OILS AND PROSTATE CANCER

Several investigators have studied the effects of omega-3 fatty acids on the risk of developing prostate cancer. Many studies have shown a reduction in the risk of developing prostate cancer when related to increased consumption of omega-3 fatty acids (Chavarro et al., 2007), whereas a more recent controversial case-cohort study by Brasky and colleagues (2013) found just the opposite: High levels of omega-3 increased the risk of developing prostate cancer by 44%. However, several concerns were raised on the overall design and methodology of this study. Reviewing the study may present more questions than answers. Should advanced practitioners advise "all men" not to take fish oil or recommend against taking omega-3 fatty acids only in "men at high risk" (positive family history)? Advanced practitioners should be prepared to discuss the potential positive and negative benefits when recommending fish oil.

## THE SELECT TRIAL

Since the publication of the SELECT trial (Selenium and Vitamin E Cancer Prevention Trial), further interest has developed in investigating "prescribing fish oil tablets" vs. "dietary intake of fish oil" in patients with prostate cancer. The SELECT trial (Lippman et al., 2009), partially funded by the National Cancer Institute and the National Center for Complementary and Alternative Medicine, was originally designed to assess the impact of several supplements and vitamins on the risk of prostate cancer. Over approximately a 3-year period, 35,533 men were randomized by study site to one of four arms: selenium and vitamin E, vitamin E and placebo, selenium and placebo, or placebo and placebo. The SELECT study was stopped early because there was no observed protective benefit in taking any of the supplements/placebos as hypothesized, nor was there any likelihood of an effect on the rates of prostate cancer in any study arm.

However, in narrowing the broad SELECT participants to a case cohort of 1,393 men, 834 of whom were diagnosed with prostate cancer, this case-cohort study found that men with higher levels of fish oil (omega-3 fatty acids) had a higher risk for developing prostate cancer (Brasky et al., 2013).

**Study Design of Plasma Phospholipid Fatty Acids**

Randomly selected from the large SELECT participants, 1,393 men were matched to case subjects based on age and race, and among them, 834 men were diagnosed with prostate cancer. Although matching was performed for the selection, there were no inclusion or exclusion criteria identified for this study. The men with prostate cancer were diagnosed by annual prostate-specific antigen (PSA) and/or digital rectal examination screening and later by pathologic reports. When possible, tissue samples were obtained, and Gleason scores were reported.

This design was rather unique in that this study was a subanalysis of the larger prospective SELECT study. The researchers analyzed patient data n that compared blood levels of omega-3 fatty acids with the incidence of cancer, which was not the original design of the SELECT study (Brasky et al., 2013). The investigators postulated that one episodic biomarker could define men’s cancer status. Also, the investigators reported that the study could not ascertain the source of omega-3 (dietary, from eating foods enriched with omega-3 such as salmon or herring, or taking fish oil supplements).

## RESULTS

The investigators performed a meta-analysis and found there was a positive association between high blood levels of omega-3 and an increased risk of developing prostate cancer. Men with the highest levels of DHA plus EPA and a third omega-3 fatty acid—docosapentaenoic acid (DPA)—were 44% more likely to develop low-grade prostate cancer (hazard ratio [HR] = 1.44; 95% confidence interval [CI] = 1.08–1.88) and 71% more likely to develop high-grade prostate cancer (HR = 1.71; 95% CI = 1.00–2.94) compared with men who had the lowest levels. The total prostate cancer risk was increased by 43% (HR = 1.43; 95% CI = 1.09–1.88). The mean percentage of total omega-3 fatty acids (EPA + DPA + DHA) was 4.66% (ranging from 4.56% to 4.75%) and 4.48% (ranging from 4.41 to 4.55, with a statistical significance of .002). Brasky and colleagues (2013) concluded that having high levels of omega-3 from fish oil increases men’s potential risk of developing prostate cancer.

## QUESTIONS REMAIN

With projections of 220,800 new cases of prostate cancer in American men in 2015 (American Cancer Society, 2015) and nearly 27,540 prostate cancer deaths, making recommendations to decrease men’s risk factors for developing prostate cancer should be a high priority for advanced practitioners. Since this study was only an observational design, causation statements were inappropriately cited in their conclusion statements. A cause-and-effect relationship remains unclear, and to conclude otherwise is not appropriate. Other researchers might say it may be coincidence, not causation.

Results of this study are compelling regarding high levels of omega-3 being found more frequently in patients with prostate cancer, but further investigation is needed to clarify what sources increase these levels. Advanced practitioners in oncology are still faced with the decision of whether to take patients off fish oil supplements or eliminate dietary intake of fish oil altogether so as not to increase their risk of prostate cancer/metastases. Although fish oil supplements continue to be of high interest for the management of several other diseases, prescribing fish oil to prevent metastases or decrease the likelihood of developing prostate cancer remains open for discussion.

## CONCLUSION

Careful analysis of the SELECT trial’s objectives is required. This trial did not have any clear study objectives regarding the relationship between omega-3 fatty acid levels and the risk of prostate cancer. The investigators were searching for any associations, found one that showed a statistical significance, and drew conclusions based on those associations. The study showed no causal link between prostate cancer and fish oil supplementation or the presence of omega-3s in the blood, yet a recommendation to avoid it was made. The study was based on serum levels of these various fatty acids and on a single blood test. The results demonstrated that there were higher levels of omega-3 fatty acids in men with prostate cancer; however, correlation in this case cohort observational study does not equal causation (Chow & Murphy, 2013). Since causation was not established, practice patterns of prescribing fish oil supplements or dietary fish oil recommendations may not change radically; rather, clinicians can begin discussions with their patients by individually looking at patient histories and what may be gained from increasing/decreasing omega-3 fatty acids.
